# Feasibility and Diagnostic Utility of Mucosal T-Cell Flow Cytometry for Intestinal Graft-Versus-Host Disease

**DOI:** 10.1016/j.gastha.2025.100820

**Published:** 2025-09-29

**Authors:** Masaya Iwamuro, Takumi Kondo, Daisuke Ennishi, Nobuharu Fujii, Mai Hiramatsu, Araki Hirabata, Takahide Takahashi, Takehiro Tanaka, Yoshinobu Maeda, Motoyuki Otsuka

**Affiliations:** 1 Department of Gastroenterology and Hepatology, Okayama University Graduate School of Medicine, Dentistry, and Pharmaceutical Sciences, Okayama, Japan; 2 Department of Hematology and Oncology, Okayama University Graduate School of Medicine, Dentistry, and Pharmaceutical Sciences, Okayama, Japan; 3 Division of Medical Support, Okayama University Hospital, Okayama, Japan; 4 Department of Pathology, Okayama University Graduate School of Medicine, Dentistry, and Pharmaceutical Sciences, Okayama, Japan

**Keywords:** cytomegalovirus infection, flow cytometry, graft-versus-host disease, hematopoietic stem cell transplantation, T lymphocytes

## Abstract

**Background and Aims:**

Timely diagnosis of intestinal complications after hematopoietic stem cell transplantation (HSCT), including graft-versus-host disease (GVHD), transplant-associated thrombotic microangiopathy, and cytomegalovirus infection, is essential for appropriate management. This study evaluated whether mucosal T-cell profiling from endoscopic biopsies could support the diagnosis of these post-transplant conditions.

**Methods:**

We prospectively analyzed 58 intestinal biopsy specimens from 21 post-HSCT patients. Paired samples were obtained from the stomach and duodenum during upper endoscopy and from the ileum and large intestine during colonoscopy. Lymphocytes were isolated from each specimen and analyzed using flow cytometry. These data were integrated with those of a previously collected cohort (35 patients, 51 samples) for comparative immunophenotypic analysis across histologically defined groups.

**Results:**

Duodenal biopsies yielded more lymphocytes than did gastric biopsies (mean ± standard deviation: 532 ± 823 vs 233 ± 392 cells; *P* = .070), with comparable yields between the ileum and colon. Among 41 evaluable cases, the CD56^+^:CD3^+^ ratio was significantly lower in patients with GVHD (5.5 ± 2.2%) than in those with nonspecific or no inflammation (28.4 ± 16.3%; *P* = .006). A cutoff value of <11% provided 85.7% sensitivity and 83.3% specificity for diagnosing GVHD (area under the curve = 0.91).

**Conclusion:**

Mucosal T-cell profiling using endoscopic biopsies is feasible and may aid in the diagnosis of GVHD after HSCT. A decreased CD56^+^:CD3^+^ ratio is a promising marker for distinguishing GVHD from other post-transplant intestinal conditions.

## Graphical abstract

**Figure:**
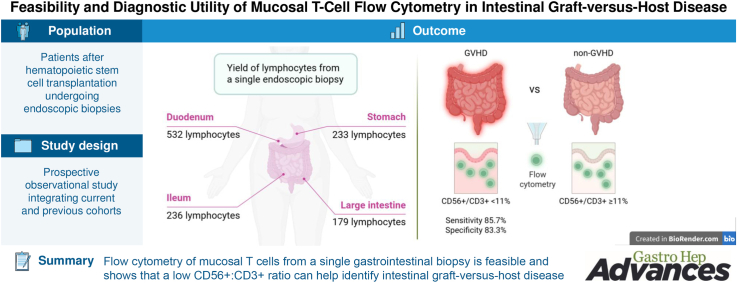

## Introduction

A variety of intestinal complications frequently arise in recipients of hematopoietic stem cell transplantation (HSCT), including acute and chronic graft-versus-host disease (GVHD), intestinal transplant-associated thrombotic microangiopathy (iTAM), and gastroenteritis or colitis caused by cytomegalovirus infection.^1^, ^2^, ^3^, ^4^, ^5^, ^6^ Flow cytometry is a powerful tool for the quantitative and multiparametric analysis of immune cell populations, enabling detailed characterization of intestinal mucosal lymphocytes. Such immunophenotypic profiling has the potential to facilitate the differential diagnosis of these overlapping post-transplant diseases, which often present with similar clinical and endoscopic findings but require distinct therapeutic approaches.

One of the major challenges in applying mucosal immunological analysis in this setting is the frequent occurrence of thrombocytopenia after HSCT, which increases the risk of bleeding associated with endoscopic biopsy procedures.^7^ Therefore, it is essential to identify intestinal sites from which sufficient numbers of lymphocytes can be reliably obtained with minimal sampling. In our previous study, we assessed the feasibility of isolating intestinal mucosal lymphocytes for flow cytometric analysis using a single endoscopic biopsy specimen obtained from the stomach, duodenum, ileum, and large intestine.^8^ We found that lymphocyte isolation was most successful in duodenal samples (8 of 9; 88.9%), followed by those from the ileum (4 of 8; 50.0%), large intestine (4 of 11; 36.4%), and stomach (8 of 23; 34.8%). These results suggest that the duodenum and ileum are more suitable than the stomach and colorectum for obtaining biopsy material for immunological analysis using flow cytometry.

Based on these findings, we conducted a prospective study in which paired mucosal biopsy samples were obtained from the stomach and duodenum during esophagogastroduodenoscopy and from the ileum and large intestine during colonoscopy. This study aimed to determine whether sufficient mucosal lymphocytes could be isolated from each of these paired sites under standard clinical conditions. Moreover, by integrating data from this new cohort with those from our previous study, we investigated whether the flow cytometric characteristics of mucosal lymphocytes could aid in distinguishing among GVHD, iTAM, and cytomegalovirus-associated enterocolitis in patients after hematopoietic stem cell transplantation.

## Methods

### Evaluation of Paired Biopsies for Lymphocyte Yield

**Figure 1:**
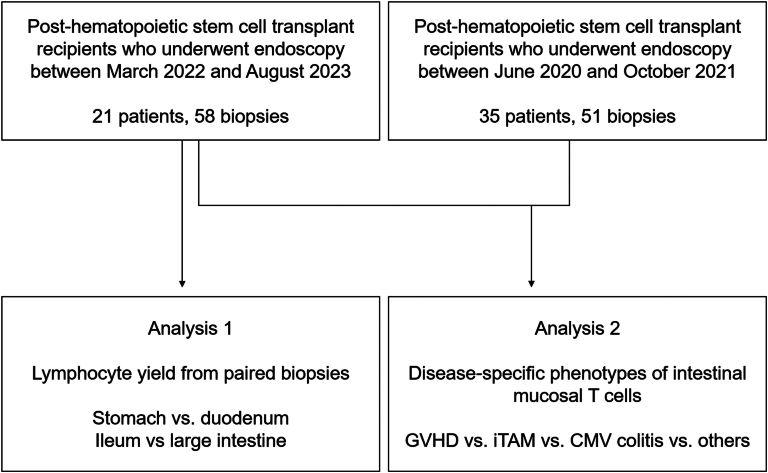
Workflow for isolation (Analysis 1) and flow cytometry (Analysis 2) of intestinal mucosal lymphocytes. In analysis 1, endoscopic biopsies were obtained from the upper (stomach and duodenum) and lower (ileum and large intestine) gastrointestinal tracts of post-transplant patients. CMV, cytomegalovirus.

Flow cytometry was prospectively performed between March 2022 and August 2023 at the Okayama University Hospital (Okayama, Japan) for 29 endoscopic procedures (esophagogastroduodenoscopy, n = 15; colonoscopy, n = 14) in 21 patients who had undergone HSCT (Figure 1). Endoscopy and biopsy were performed as part of standard care when investigating abdominal symptoms such as pain, diarrhea, epigastralgia, and appetite loss, or as surveillance of the gastrointestinal tract for GVHD. Using disposable biopsy forceps, a single specimen was obtained from the stomach and duodenum during esophagogastroduodenoscopy and from the ileum and large intestine during colonoscopy.

Lymphocytes were isolated from each specimen using a previously reported one-step lymphocyte isolation procedure.^9^^,^^10^ Sample acquisition and lymphocyte isolation were performed by an endoscopist (M.I.). Lymphocytes isolated from the gastrointestinal mucosa were subjected to flow cytometry. Lymphocyte populations were identified and quantified using CD45 expression in combination with side scatter and forward scatter integral parameters. Lymphocyte enumeration was independently conducted by clinical laboratory technologists (M.H., A.H., and T.T.) who were blinded to all the clinical data. We compared the number of isolated lymphocytes between the stomach and duodenum, as well as between the ileum and the large intestine (cecum, colon, or rectum).

### Analysis of Disease-specific Phenotypes of Intestinal Mucosal T Cells

To comprehensively analyze the immunophenotypic profiles of intestinal T lymphocytes in patients after HSCT, we integrated the flow cytometry datasets obtained from the present study (conducted between March 2022 and August 2023) with those from our previous investigation (conducted between June 2020 and October 2021). Patients were categorized into 4 groups based on histopathological findings: intestinal GVHD, iTAM, gastrointestinal cytomegalovirus infection, and others (including histologically normal mucosa and nonspecific inflammation). Patients with fewer than 50 isolated lymphocytes were excluded from the analysis. When multiple flow cytometry assessments were performed on the same patient, the dataset with the highest number of isolated lymphocytes was selected for evaluation.

**Figure 2:**
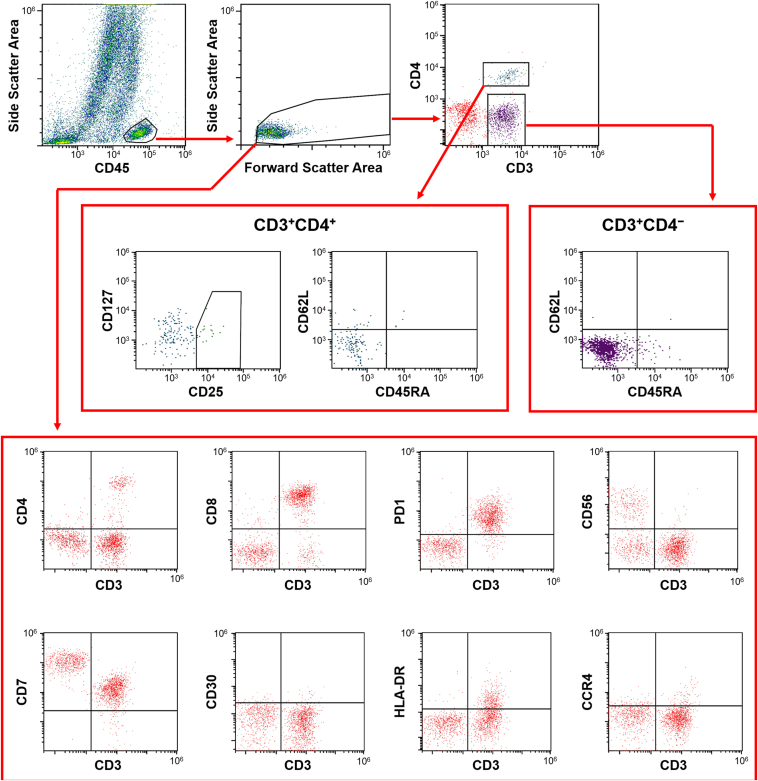
Gating strategy used in this study. Lymphocyte populations were identified and quantified based on CD45 expression combined with side and forward scatter integral parameters. Surface markers including CD4, CD8, PD-1, CD56, CD7, CD30, HLA-DR, and CCR4 were analyzed. Lymphocytes were separated into CD3^+^CD4^+^ and CD3^+^CD4^-^ populations, and CD45RA and CD62L expression levels were evaluated to classify lymphocytes into functional subsets such as naïve T cells, central memory T cells, and effector memory T cells. The proportion of regulatory T cells within the CD3^+^CD4^+^ population was assessed based on CD25 and CD127 expression.

We analyzed the expression of surface markers including CD4 (helper T lymphocytes), CD8 (cytotoxic T lymphocytes), CD25 and CD127 (regulatory T cells), CD56 (natural killer cells; also known as neural cell adhesion molecule), CD7 (mature T lymphocytes), PD-1 (CD279) (central inhibitory receptor), CD30 (activated T lymphocytes; also known as TNFRSF8), HLA-DR (human leukocyte antigen DR isotype), and CCR4 (CD194) (Th2 marker). Additionally, CD45RA and CD62L expression levels were evaluated to classify lymphocytes into functional subsets such as naïve T cells, central memory T cells, and effector memory T cells. The gating strategy employed in this study is depicted in Figure 2.

### Statistical Analysis

Statistical analyses were performed using JMP (version 14.0.0; SAS Institute Inc., Cary, NC). Paired t-tests were performed to compare lymphocyte yields between the stomach and duodenum and between the ileum and large intestine. Student’s t-test or F-test was used to compare the two population means. For multiple comparisons, statistical analysis was performed using 1-way analysis of variance followed by the Tukey-Kramer post-hoc test. Statistical significance was set at *P* < .05. Numerical values are presented as means ± standard deviations (SDs).

## Results

### Patient Characteristics

The clinical characteristics of the enrolled patients are summarized in Table 1. This study included 13 men and 8 women. The average age at the initial flow cytometry examination for gastrointestinal tract-resident lymphocytes was 50 years (range: 20–70 years). The median interval between transplantation and endoscopy was 40 days (range: 19–513 days). The underlying hematological diseases included acute myeloid leukemia (n = 10), acute lymphocytic leukemia (n = 3), myelodysplastic syndrome (n = 3), chronic myelogenous leukemia (n = 2), primary central nervous system diffuse large B-cell lymphoma (n = 2), angioimmunoblastic T-cell lymphoma (n = 2), and adult T-cell leukemia/lymphoma (n = 2). Patients underwent allogeneic peripheral blood stem cell transplantation (n = 12), cord blood transplantation (n = 7), or bone marrow transplantation (n = 2). The pathological diagnoses were nonspecific or no inflammation (n = 13), intestinal GVHD (n = 4), and iTAM (n = 4). No patients with gastrointestinal cytomegalovirus infections were identified in this study.

**Table 1:** Patient Characteristics

Characteristic	n
Sex	
Male	13
Female	8
Age (y), median (range)	59 (20–70)
Days after SCT, median (range)	40 (19–513)
Underlying diseases	
Acute myeloid leukemia	10
Acute lymphocytic leukemia	3
Myelodysplastic syndrome	3
Chronic myelogenous leukemia	2
Primary central nervous system diffuse large B-cell lymphoma	2
Angioimmunoblastic T-cell lymphoma	2
Adult T-cell leukemia/lymphoma	2
Type of transplantation	
Allogeneic peripheral blood SCT	12
Cord blood transplantation	7
Bone marrow transplantation	2
Pathological diagnosis	
Nonspecific or no inflammation	13
Graft-versus-host disease	4
iTAM	4

SCT, stem cell transplantation.

Regarding the procedures, 7 patients underwent only esophagogastroduodenoscopy, another 7 underwent only colonoscopy, and 4 underwent both procedures once. One patient underwent an esophagogastroduodenoscopy twice and a colonoscopy once, whereas the other underwent 2 colonoscopies. Additionally, one patient underwent esophagogastroduodenoscopy twice. Fifteen and 14 esophagogastroduodenoscopies and colonoscopies were performed, respectively.

**Figure 3:**
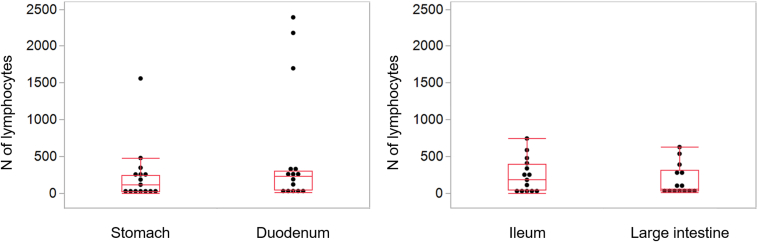
Lymphocyte yields from biopsy sites in the gastrointestinal tract. Lymphocyte yields were compared among the different biopsy sites. Duodenal biopsies showed a trend toward higher lymphocyte yields than did gastric biopsies, although the difference was not statistically significant (*P* = .070). No significant differences were observed between ileal and colonic samples. Data are presented as mean ± standard error.

The number of isolated lymphocytes tended to be higher in the duodenum (mean ± SD: 532 ± 823) than in the stomach (233 ± 392), although the difference did not reach statistical significance (*P* = .070, paired t-test) (Figure 3). Conversely, the number of isolated lymphocytes in the ileum (236 ± 230) was comparable to that in the large intestine (179 ± 208), with no significant difference (*P* = .331, paired t-test). The proportion of biopsy samples from which more than 50 lymphocytes per biopsy were successfully isolated was 60% (9 of 15 cases) in the stomach and 83% (11 of 15 cases) in the duodenum, with no statistically significant difference observed between the 2 sites (*P* = .700). Similarly, no significant difference was observed in yield between the ileum (11 of 14 cases, 78.6%) and the large intestine (8 of 14 cases, 57.1%) (*P* = .261).

### Characteristics of Gastrointestinal Tract-Resident Lymphocytes in Stem Cell Transplant Recipients and Their Association with the Pathological Diagnosis

**Figure 4:**
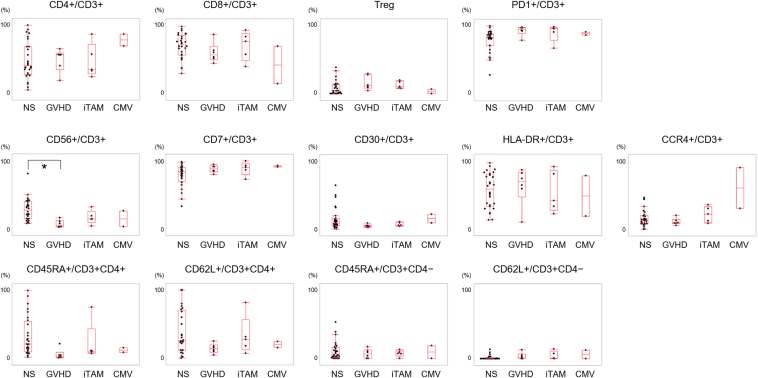
Flow cytometry analysis of T lymphocytes in the intestinal mucosa among disease groups. The ratio was evaluated across histologically defined disease groups: no or NS, GVHD, iTAM, and CMV. Regulatory T (Treg) cells were defined as CD3^+^CD4^+^CD25^+^CD127^low/−^ cells. Patients with CMV infection (n = 2) were excluded from statistical analysis due to the small sample size. ∗, *P* < .01. CMV, cytomegalovirus infection; NS, nonspecific inflammation.

We combined the data from the current cohort with those from our previous study (51 samples, 35 patients),^8^ resulting in 115 samples from 57 patients. After excluding samples with < 50 lymphocytes (n = 41) and duplicate samples from the same patient (n = 33), 41 samples were included in the comparative analysis. The diagnoses for these samples comprised nonspecific or no inflammation (n = 28), GVHD (n = 6), iTAM (n = 5), or cytomegalovirus infection (n = 2). Owing to the limited number of cytomegalovirus cases, these are shown in Figure 4 for reference but excluded from statistical comparisons.

The flow cytometry results are shown in Figure 4. The CD56^+^:CD3^+^ ratio was significantly lower in the GVHD group (mean ± SD: 5.5 ± 2.2%) than in the group with nonspecific or no inflammation (28.4 ± 16.3%, *P* = .006). No other immunophenotypic markers demonstrated significant differences between groups.

**Figure 5:**
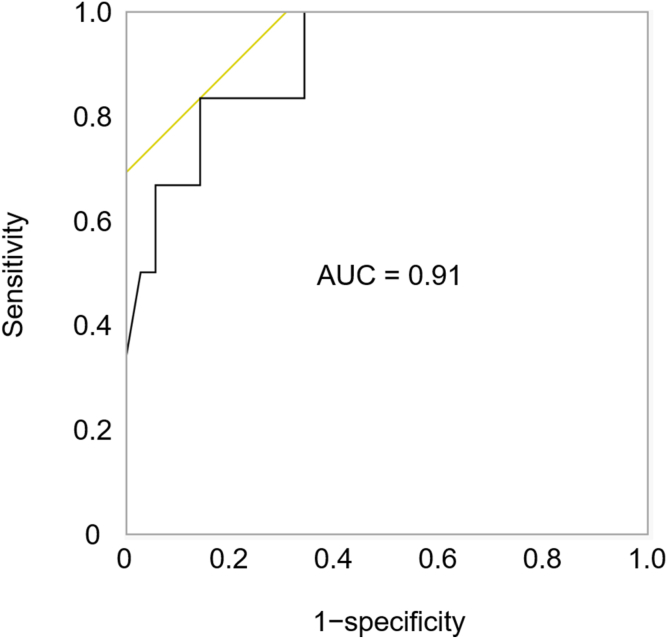
Diagnostic performance of CD56^+^:CD3^+^ ratio for intestinal GVHD. ROC curve for distinguishing GVHD from non-GVHD using the CD56^+^:CD3^+^ ratio. A cutoff value of <11% yielded 85.7% sensitivity and 83.3% specificity for the diagnosis of GVHD (AUC = 0.91). AUC, area under the curve; ROC, receiver operating characteristic.

To further evaluate the diagnostic value of the CD56^+^:CD3^+^ ratio for GVHD, we performed receiver operating characteristic curve analysis to compare the GVHD and non-GVHD groups (the latter comprising iTAM, cytomegalovirus, and nonspecific inflammation). The area under the curve was 0.91. A cutoff value of <11% for the CD56^+^:CD3^+^ ratio yielded a sensitivity of 85.7% and specificity of 83.3% for the diagnosis of GVHD (Figure 5).

## Discussion

In this prospective study involving paired mucosal biopsies, we evaluated the feasibility of isolating sufficient lymphocytes from various gastrointestinal sites in post-transplant patients and assessed the potential utility of flow cytometric analysis for differential diagnosis. Although the number of isolated lymphocytes did not differ significantly between the stomach and duodenum or between the ileum and large intestine, a trend toward higher yields from the duodenal samples was observed. These findings support the practicality of obtaining a single duodenal biopsy specimen for flow cytometric analysis under routine clinical conditions. This approach may help optimize sample quality while minimizing procedural risks in patients with post-transplant thrombocytopenia. Conversely, the lymphocyte yields from the ileum and large intestine were comparable, suggesting that either site could be flexibly selected during colonoscopy.

By integrating data from this cohort with those from our previous study,^8^ we also investigated whether the immunophenotypic features of mucosal lymphocytes could aid in distinguishing GVHD from other post-transplant intestinal complications. Among the analyzed markers, the CD56^+^:CD3^+^ ratio was significantly lower in the GVHD group than in the non-GVHD group. While CD3^+^CD56^+^ cells have conventionally been referred to as "natural killer T (NKT) cells," this population actually represents a heterogeneous group, including activated T cells, natural killer (NK)-like T cells, and only a minority of true invariant NKT (iNKT) cells.^11^^,^^12^ Therefore, CD3^+^CD56^+^ cells are more appropriately described as NK or NKT-like cells.^13^, ^14^, ^15^ Functionally, NKT-like cells are known to exert immunomodulatory effects, including cytokine production, expansion of regulatory T cells, and suppression of antigen-presenting cells. These mechanisms contribute to the regulation of alloreactive T-cell responses and the maintenance of immune homeostasis following HSCT. The observed reduction of CD3^+^CD56^+^ cells in patients with GVHD may reflect a loss of these regulatory functions within the mucosal immune system, potentially exacerbating inflammation and tissue damage characteristic of GVHD. Studies have shown that iNKT cell numbers are reduced in patients with both acute and chronic GVHD.^16^ Notably, the identification of iNKT cells requires CD1d tetramer staining, which is beyond the scope of this study. Therefore, further investigations are needed to elucidate the role of CD3^+^CD56^+^ cells, the so-called “NKT-like cells,” in intestinal GVHD.

From a diagnostic perspective, receiver operating characteristic curve analysis demonstrated that a CD56^+^:CD3^+^ ratio below 11% had high sensitivity and specificity for the diagnosis of GVHD, suggesting that quantification of these cells in intestinal mucosa could serve as a useful biomarker. This finding supports the potential clinical utility of mucosal immunophenotyping in the differential diagnosis of post-transplant complications.

This study had some limitations. The sample size for each disease group, particularly for GVHD, iTAM, and cytomegalovirus infection, was relatively small, which may constrain the generalizability of our results. Additionally, due to the exclusion of low-cell-yield samples, there exists a potential for selection bias. Further validation in larger multicenter cohorts and longitudinal analyses is imperative to confirm the robustness and clinical applicability of our findings.

## Conclusion

Flow cytometry analysis of mucosal lymphocytes is feasible using single endoscopic biopsies, particularly from the duodenum, and may offer valuable immunological information to support the diagnosis of GVHD. The CD56^+^/CD3^+^ ratio is a promising biomarker for identifying intestinal GVHD and may contribute to improved diagnostic precision and personalized management of HSCT recipients.
